# Distinct DNA methylation patterns of cognitive impairment and trisomy 21 in down syndrome

**DOI:** 10.1186/1755-8794-6-58

**Published:** 2013-12-27

**Authors:** Meaghan J Jones, Pau Farré, Lisa M McEwen, Julia L MacIsaac, Kim Watt, Sarah M Neumann, Eldon Emberly, Max S Cynader, Naznin Virji-Babul, Michael S Kobor

**Affiliations:** 1 Centre for Molecular Medicine and Therapeutics, Child and Family Research Institute, and Department of Medical Genetics, University of British Columbia, Vancouver, British Columbia, Canada; 2 Department of Physics, Simon Fraser University, Burnaby, British Columbia, Canada; 3 Department of Psychology, Simon Fraser University, Burnaby, British Columbia, Canada; 4 Brain Research Centre, University of British Columbia, Vancouver, British Columbia, Canada; 5 Department of Physical Therapy, University of British Columbia, Vancouver, British Columbia, Canada; 6 Human Early Learning Partnership, School of Population and Public Health, University of British Columbia, Vancouver, British Columbia, Canada

**Keywords:** Down syndrome, DNA methylation, Cognitive impairment, Aging, Illumina 450k human methylation array

## Abstract

**Background:**

The presence of an extra whole or part of chromosome 21 in people with Down syndrome (DS) is associated with multiple neurological changes, including pathological aging that often meets the criteria for Alzheimer’s Disease (AD). In addition, trisomies have been shown to disrupt normal epigenetic marks across the genome, perhaps in response to changes in gene dosage. We hypothesized that trisomy 21 would result in global epigenetic changes across all participants, and that DS patients with cognitive impairment would show an additional epigenetic signature.

**Methods:**

We therefore examined whole-genome DNA methylation in buccal epithelial cells of 10 adults with DS and 10 controls to determine whether patterns of DNA methylation were correlated with DS and/or cognitive impairment. In addition we examined DNA methylation at the *APP* gene itself, to see whether there were changes in DNA methylation in this population. Using the Illumina Infinium 450 K Human Methylation Array, we examined more than 485,000 CpG sites distributed across the genome in buccal epithelial cells.

**Results:**

We found 3300 CpGs to be differentially methylated between the groups, including 495 CpGs that overlap with clusters of differentially methylated probes. In addition, we found 5 probes that were correlated with cognitive function including two probes in the *TSC2* gene that has previously been associated with Alzheimer’s disease pathology. We found no enrichment on chromosome 21 in either case, and targeted analysis of the *APP* gene revealed weak evidence for epigenetic impacts related to the AD phenotype.

**Conclusions:**

Overall, our results indicated that both Trisomy 21 and cognitive impairment were associated with distinct patterns of DNA methylation.

## Background

Down syndrome (DS) occurs in approximately 1 out of every 600 live births in the US and is the most prevalent genetic cause of developmental disabilities [1]. It is due to the presence of an additional whole or partial copy of chromosome 21 resulting in developmental changes beginning early in fetal life. Clinical features of DS include mental retardation, stereotypical facial features, poor muscle tone, and short stature. People with DS are at increased risk of congenital heart disease, periodontal disease, diabetes and leukemia, and often show accelerated cognitive impairment with age [2-4].

Postmortem studies show that from the age of 40 upward, individuals with DS are at much higher risk than the general population of having neuropathological changes that meet the clinical criteria for Alzheimer’s Disease (AD) [5-7]. These include extensive cerebral atrophy, accumulation of β-amyloid, extracellular senile plaques and intracellular neurofibrillary tangles in the hippocampus, and frontal and temporal cortices. In addition, functional brain imaging studies reveals spectral slowing in the brain activity of DS subjects, particularly in bilateral temporal regions known to be associated with learning and memory [8]. Memory impairments are hypothesized to be associated with the amyloid precursor protein gene on chromosome 21 [5,6,9]. This gene is thought to have multiple possible implications to the etiology of DS which overlap with AD symptoms, including transcriptional modulation and amyloid plaque formation [8,10,11]. Since individuals with DS have three copies of chromosome 21, it is suspected that an overexpression of the amyloid precursor protein contributes to the increased risk of AD in this population [7,12,13].

One mechanism by which cells may respond to changes in gene dosage is altered DNA methylation. DNA methylation is one of a group of epigenetic modifications to the genome which affect the ability of specific genes to be expressed but do not modify the sequence of the genome itself. One of the best-characterized effects of DNA methylation is its contribution to the inactivation of one entire X chromosome in females, which restores dosage equality with XY males. Methyl groups are added to CpG dinucleotides, and these modifications in turn recruit chromatin remodeling complexes which alter the structure of the surrounding chromatin, either increasing or decreasing the availability for the gene to be expressed. Changes in DNA methylation are associated with both normal aging and with Alzheimer’s Disease [14-16]. Additionally, the *APP* promoter is specifically hypomethylated in brain tissues from AD patients [17]. Previous studies examined leukocytes and fetal tissues of DS participants for changes in DNA methylation using lower-resolution genome-wide approaches, and found significant differences in a number of genes, as did a recent study examining DS placental tissue using reduced representation bisulfite sequencing [18-20].

We hypothesize that altered DNA methylation on chromosome 21 and across the genome may be associated with accelerated cognitive aging in DS. To that end, we examined a cohort of 10 adult participants with DS and 10 age- and sex-matched controls. We evaluated cognitive function and collected cheek swabs from each participant. Genome-wide DNA methylation patterns were analyzed using the Illumina 450K Human Methylation Array, which interrogates over 480,000 CpG dinucleotides in the genome, including over 4000 on Chromosome 21 itself, and were correlated with scores related to cognitive function.

## Methods

### Participants

This study was approved by the University of British Columbia Clinical Research Ethics Board. Participants with DS were recruited from the Down Syndrome Research Foundation (DSRF), in Burnaby, B.C. Informed consent was provided by either a parent or guardian, and assent was obtained from the participant. Age and gender matched control participants were recruited from the staff and students at the Child and Family Research Institute (CFRI) in Vancouver, B.C. A total of 20 adults between the ages of 27–46 years of age, 10 with DS (5 Male, 5 Female) and 10 controls (5 Male, 5 Female) participated in this study. All participants were financially compensated for parking and travel costs.

### Dalton brief praxis test

The Dalton Brief Praxis Test (BPT) is an abbreviated, 20-item version of the Dyspraxia Scale for Adults with Down’s Syndrome, a 62-item cognitive test of praxis. It scores the ability to perform simple, highly practiced, voluntary movements in response to a verbal command or imitation, and therefore measures verbal comprehension and motor co-ordination and is used to monitor changes in cognitive function. The BPT was administered by a trained research assistant.

### DNA isolation and DNA methylation arrays

Buccal (cheek) swabs were collected from each participant using standard protocols, and DNA isolated using the Isohelix Buccal DNA isolation kit (Cell Projects, Kent, UK) as per manufacturer’s instructions. DNA was then purified and concentrated using the DNA Clean & Concentrator kit (Zymo Research, Irvine, CA, USA). Approximately 750 ng of DNA was used for bisulfite conversion using the Zymo Research EZ DNA Methylation Kit (Zymo Research, Irvine, CA, USA). Sample yield and purity was assessed after each step using a Nanodrop ND-1000 (Thermo Scientific, Irvine, CA). After bisulfite conversion, 160 ng of DNA was applied to the Illumina 450 K methylation array, as per manufacturer’s protocols (Illumina, San Diego, CA, USA).

### Data quality control and normalization

Data was subjected to stringent quality control before being normalized in R [21]. First, probes for which detection p-values were greater that 0.01, probes with missing beta values, and probes for which less than three beads contributed to the signal were eliminated in any sample (a total of 8620). Next, 11,648 probes on the X or Y chromosome and 65 probes examining single nucleotide polymorphisms were removed from further analysis. More recent annotation of the Human Methylation 450 k array was used to filter 32,494 probes that are known to be polymorphic at the CpG, or probes which have *in silico* nonspecific binding to the X or Y chromosomes [22]. Together, these measures eliminated 52,827 probes, leaving a total of 432,750 probes for further analysis. Raw data has been deposited in GEO, accession number GSE50586. Colour correction, background adjustment, and quantile normalization were performed using the lumi R package, and data was normalized using peak-based correction [23]. ComBat was used to remove any effects of batch from our data [24,25]. Correlations for two technical replicates were 0.9949 and 0.9939 before ComBat, and 0.9973 and 0.9963 after ComBat, indicating minimal batch effect, which was nonetheless corrected.

### Principal component analysis

Principal Component Analysis (PCA) decomposes the measured methylation patterns into a set of linearly independent principal component (PC) patterns that are ranked according to how much variance in the data they explain. The methylation pattern of each probe *i* across all samples, $\vec{x_{i}}$, can be written as $\vec{x_{i}}=\bar{x}+\sum_{j}a_{\mathrm{ij}}\vec{v_{j}}$ where $\bar{x}$ is the mean profile calculated over all the probes in the dataset. $\vec{v_{j}}$ are the eigenvectors (PCs), and *a*_*ij*_ are the projection values of each probe *i* onto the eigenvector *j*. The top ranked PCs can often be correlated with known traits in the cohort such as tissue type, cellular composition, or disease state. Because PCs are linearly independent, a particular PC’s contribution to each probe’s methylation pattern can be subtracted out without altering the information contained in the pattern arising from all the others. For this dataset, PCA was performed on the normalized data set twice; first on the 10 DS and 10 control samples and a second time with ten additional blood samples added. These blood samples were from unrelated healthy individuals of the same approximate ages. As described in more detail in the result section, the initial PCA without the blood samples revealed an unusual clustering of samples in the first PC, possibly indicating blood contamination of the buccal swabs. The second PCA including blood samples showed that indeed, some of our buccal swabs had scores more similar to blood than other buccal swabs for PC1. Since the dependency of methylation on tissue represented by that PC1 for this study is a confounder, its contribution to each probe was subtracted out, yielding methylation data that no longer has variation due to tissue differences. A dataset $\vec{x_{i}^{*}}$ were the contribution of PC number *k* is subtracted out is constructed as $\vec{x_{i}^{*}}=\bar{x}+\sum_{j\neq k}a_{\mathrm{ij}}\vec{v_{j}}$. Our final data set then had 388,607 probes, since only probes for which we had data for all samples – the 20 from our DS/Control study plus the ten blood samples we used to determine the tissue-related variation in our data. Dendrograms were generated using Euclidean distance.

### Differential methylation analysis

All statistical analysis on normalized and corrected data was performed using R statistical software (version 3.0). Probes with DNA methylation levels significantly different between DS and control participants were identified first using the R limma package’s moderated t-tests with empirical Bayesian variance method and Benjamini-Hochberg correction to control the false discovery rate at 0.01 [26]. Significant probes were then filtered to include only those that also had a beta value difference between DS and control group means (∆beta) of at least 10%. This cutoff is used to eliminate probes that have relatively small magnitude of change between groups regardless of their statistical significance. In addition, we used the “Bump Hunting” method from the R CHARM package to discover groups of probes that show differential methylation, and used this list of differentially methylated regions (DMRs) to identify genes that contain multiple differentially methylated probes [27].

Correlations between BPT scores in DS participants and DNA methylation levels both for all probes and for the *APP* probes specifically were performed in R using a 2-sided Spearman correlation, and p-values were corrected with Benjamini-Hochberg or Bonferroni correction, as noted. For whole-genome correlations, probes were considered significantly correlated with BPT if the Benjamini-Hochberg corrected p-values were below 0.01 and the range of highest to lowest beta values was greater than 10%. T-test were performed using the base *t*-test function in R [21].

All statistical analysis was performed on transformed M-values [28]. All values given in figures and the text are expressed as beta values.

### DAVID analysis

Significant probe accession names were input into the DAVID online GO clustering tool [29]. Only the probes used in this analysis from the Illumina 450 k Human Methylation array were used as a background list. Consistent with published approaches, clusters with enrichment scores greater than 1.3 were considered significant [29].

## Results

### Participants and brief praxis scores

Scores on the BPT ranged from 35 to 80 for the 10 participants with DS, and all 10 control participants received a score of 100 (Table 1). Mean age and SD were matched across groups (means 34.13 for DS and 34.5 for control, SD 6.12 for DS and 6.78 for control), and a *t*-test p-value of 0.90 showed good matching of cohort ages. Praxis scores were not significantly correlated with age in the DS cohort (Spearman’s correlation p = 0.55).

**Table 1:** Demographics and brief praxis scores of all participants

**Sample ID**	**Sample group**	**Sex**	**Age at time of sampling**	**Total brief praxis score**
C1	Control	F	30.00	100
C2	Control	M	28.00	100
C3	Control	F	45.00	100
C4	Control	M	47.00	100
C5	Control	M	33.00	100
C6	Control	F	30.00	100
C7	Control	F	38.00	100
C8	Control	M	30.00	100
C9	Control	M	29.00	100
C10	Control	F	35.00	100
DS01	DS	M	46.56	35
DS02	DS	F	38.40	80
DS03	DS	F	30.27	80
DS04	DS	F	40.68	74
DS05	DS	F	35.69	76
DS06	DS	M	29.55	70
DS07	DS	M	29.52	80
DS08	DS	F	30.38	78
DS09	DS	M	32.96	64
DS10	DS	M	27.29	67

### Principal component analysis to eliminate sample tissue variability

**Figure 1:**
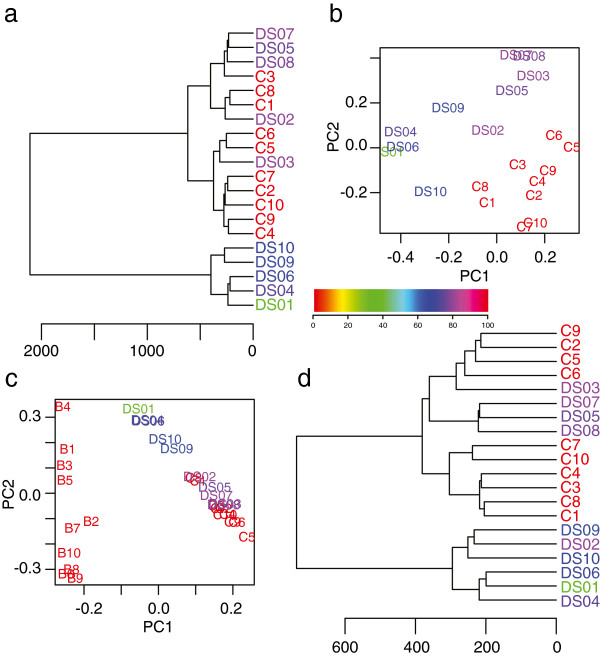
**Clustering and Principal Component analysis revealed and removed contaminating epigenetic signatures from blood in buccal swabs. a)** Dendrogram of relatedness of overall patterns of DNA methylation in all 20 individuals studied. In all figure parts, colours of participant codes indicate Praxis scores, as shown in scale in centre. **b)** PCA plot showing individual participants’ scores for PC1 (x-axis) and PC2 (y-axis) without correction. **c)** PCA plot showing scores for PC1 and PC2 for individual participants as well as 10 blood samples. Five DS participants clearly scored intermediate between the other buccal samples and the blood scores for PC1. **d)** Dendrogram of relatedness of overall patterns of DNA methylation in all 20 individuals studied after removal of PC1 from **c**. Overall distances between samples were reduced.

Hierarchical clustering of global DNA methylation placed the 5 DS cases with the higher BPT scores closer to the controls than participants with lower scores though the difference in scores between the groups was not statistically significant (Welsh two sample t test p value = 0.07) (Figure 1a). Age was also not significantly different between the groups with high and low BPT scores (Welsh two sample t test p value = 0.55).

Principal component analysis (PCA) was used to determine the sources of variance across all samples and probes. Initial PCA revealed that the 5 participants with low BPT scores had markedly different scores for the first PC, which accounted for 64.8% of the variation, when compared to controls or the remaining DS participants (Figure 1b). This amount of variance was unlikely to be due simply to differences in cognitive impairment, since previous studies using PCA have generally identified the first PC as being associated with either tissue differences or probe-to-probe variation [30,31]. Given that people with DS are at increased risk of periodontal disease, we were concerned about blood contamination in our collected buccal swabs [4]. We added 10 unrelated blood samples to our PCA and repeated the analysis, showing that the new first PC, which had a very similar shape to the previous first PC, separated tissue types (Figure 1c). In particular it segregated blood from buccal samples, with the low BPT scoring DS participants having intermediate scores. This could indicate that the different scores in the original PCA between the low and high BPT were due to the five low BPT buccal swabs having become contaminated with a small amount of blood. This could be due to the participants having bitten their cheeks or tongue, or may simply be due to thinner epithelium and greater probability of periodontal disease in DS participants [4].

In order to analyze the true differences between the DS participants and controls, as well as the differences correlated with BPT, it was important to eliminate this possible cell type-related confounder. To control for this, we subtracted from our data set the effects of PC1 from the PCA that included the blood samples (see Methods). This eliminated the variance due to tissue contamination while leaving any real DNA methylation differences between DS and control and any correlations with cognitive impairment. After subtraction we repeated the hierarchical clustering and noted that the distance between samples was reduced, reflecting the reduced variation in the modified data set (Figure 1d). The cluster for the non-contaminated samples changed as well, likely due to the reduction in variance from all samples; any variance associated with the blood/buccal difference would be eliminated, and so the noise that had been obscuring the true relationship between the samples was removed. The five low BPT samples now clustered with one of the other DS samples, which could be due either to a small remnant of the blood differences in the data, or a true difference between the groups. The subtracted data was a significant improvement over the original. After the PC1 subtraction, the new PC1 accounted for a total of 24.1% of the variance and was correlated with both DS and BPT score, and PC2, accounting for 11.2% of the variance, was correlated with DS (Additional file 1: Table S1).

### Differential DNA methylation between DS and controls

**Figure 2:**
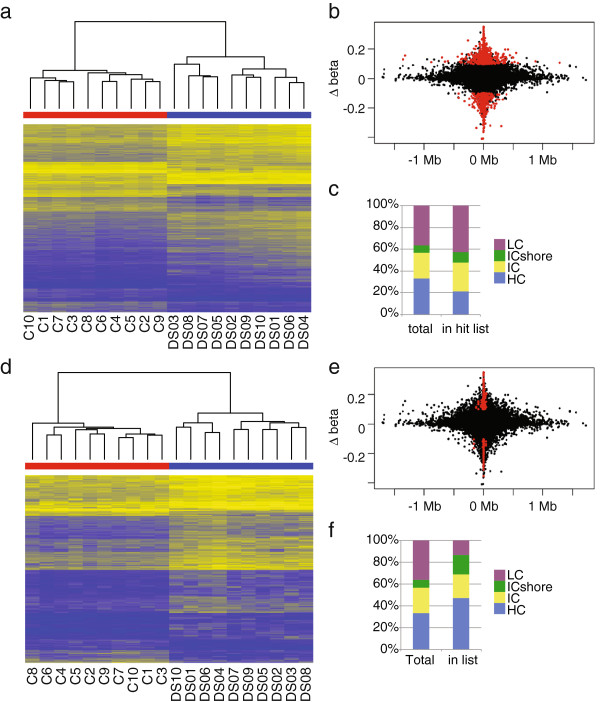
**Epigenetic signature of T21. a)** Heatmap of beta values of probes that were significantly different between DS and control participants with a difference between the means of the groups >10%, 3300 probes total. Yellow indicates higher methylation, and blue indicates lower methylation. DS participants are shown in blue, controls in red. **b)** Scatterplot showing relationship of ∆beta (mean difference in beta value between DS and control participants, y-axis) and distance to transcriptional start site (TSS, x-axis). Probes in red are significantly differently methylated between DS and control as in **a**. **c)** Breakdown of CpG island type in entire array (left column) and significantly different probes shown in **a** (right column). **d)** Heatmap of beta values of probes from **a** that also overlap with regions of differential methylation identified using the “Bump Hunter” method, 495 total. **e)** Scatterplot showing relationship of ∆beta (mean difference in beta value between DS and control participants, y-axis) and distance to transcriptional start site (TSS, x-axis). Probes in red are significantly differently methylated between DS and control as in **d**. **f)** Breakdown of CpG island type in entire array (left column) and significantly different probes shown in **d** (right column).

Using a linear model fitting method, we found 9,982 probes that were significantly different between DS and control samples after Benjamini-Hochberg correction. We then calculated the absolute difference between means of beta values for DS and controls, and refer to it as Δbeta. Of the 9,982 significant probes, 3300 had a Δbeta of more than 0.1, meaning that the mean methylation values between groups are different by more than 10%, which we refer to as differentially methylated probes (DMPs, Figure 2a, Additional file 2: Table S2). Of these, 2,190 were more methylated in DS and 1,110 were more methylated in controls. DMPs were distributed up to 1 Mb away from the nearest TSS, though hits with higher ∆beta values tended to be found closer to TSS sites (Figure 2b). CpG island distribution was significantly altered from the array background (chi-square pval < 0.005, Figure 2c), with high-density CpG islands (HC) depleted and low-density islands enriched, while IC and ICshore proportions were similar. Finally, we found no enrichment or depletion for sites mapping to chromosome 21 (Fisher two-sided exact test p = 0.09).

To determine whether we had multiple hits per gene, we used the Bump Hunter function in the CHARM R package to find differentially methylated regions (DMRs) [27]. We found 495 of our DMPs were also found in Bump Hunter clusters (BH-DMPs) (Figure 2d, Additional file 3: Table S3). BH-DMPs tended to be found very close to the TSS (Figure 2e), and island distribution was also significantly altered from the array background (chi-square pval < 0.005, Figure 2f).

Within the DMPs and BH-DMPs, we found a number of genes known to have functions related to the pathology of DS. *TFAP2B* had 26 out of a total of 45 probes on the array significantly different between DS and control, 13 of which overlapped with Bump Hunter clusters, and all of which were more methylated in DS by a range of 10-35%. The significant probes are located between 1.5 kb upstream of the TSS to 8 kb downstream. This gene was found in a GWAS study to be associated with type 2 diabetes, and was further characterized to be involved in glucose uptake and insulin resistance in adipocytes [32,33]. *DLX5* and its near neighbor *DLX6-AS* had a total of 42 probes in the DMPs, with 28 of these in the BH-DMPs. *DLX5* and *DLX6* have been identified as being important in neural crest differentiation, including GABAergic neurons of the developing forebrain and craniofacial development, particularly jaw development [34,35]. *TNXB* has 23 probes significantly different between DS and control, all of which are also found in the BH-DMPs. This gene is an extracellular matrix protein responsible for Ehlers-Danos syndrome, which has been hypothesized to have clinical overlap with DS [36]. Finally, *CPT1B* with 12 CpGs in the DMP and BH-DMP lists, is a carnitine palmitoyltransferase specifically expressed in mitochondria of skeletal muscle and associated with metabolic syndrome and lipid deposition [37,38]. Reflecting these interesting hits with clear linkages to the biology of DS and AD, DAVID analysis of our DMP list showed 17 significant clusters of GO terms (Additional file 4: Table S4). The top cluster had an enrichment score of 8.05 and was related to cell adhesion, the second had an enrichment score of 3.85 and included terms related to protein phosphorylation. The third cluster had a score of 2.46 and was centered on neural development and differentiation. Our list of DMPs that overlap with Bump Hunter clusters did not reveal any significant DAVID enrichment categories.

### Specific CpGs correlated with cognitive function

**Figure 3:**
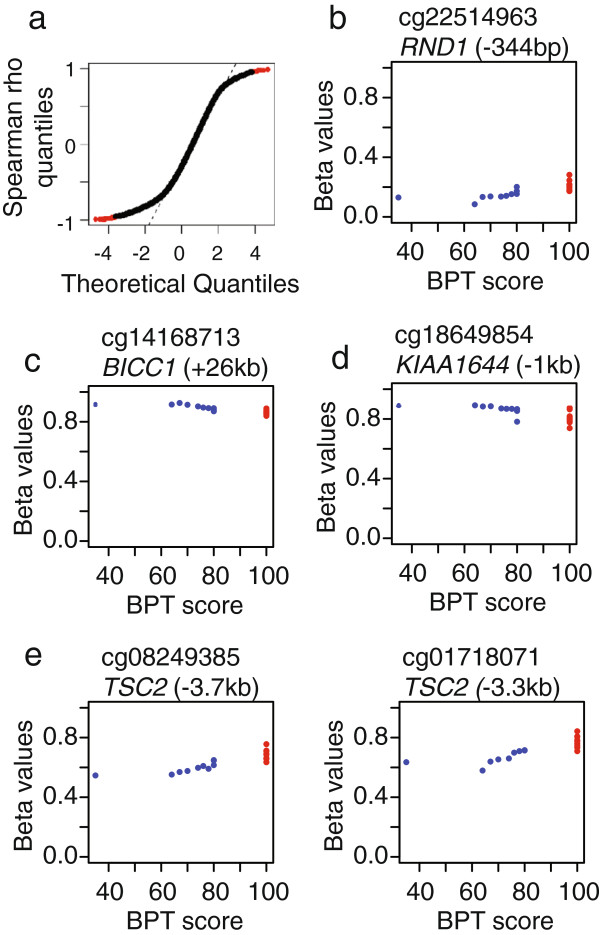
**DNA methylation sites correlated with cognitive impairment in DS participants. a)** Normal Q-Q plot of Spearman rho correlation coefficients for all probes. S-shape indicates small tails on the distribution of coefficients. Black points are non-significant probes, red points are probes which survive Benjamini-Hochberg correction for correlation between DNA methylation and BPT in DS participants. **b**-**e**) BPT scores (x-axis) plotted against beta values (y-axis) for five probes found to be significantly correlated with BPT in DS participants and significantly different between DS and controls. Blue points indicate DS participants and red points indicate controls. **b)***RND1***c)***BICC1***d)***KIAA1644* and **e)** Two probes in *TSC2*.

For correlations between BPT scores in DS participants and DNA methylation, we used two approaches. First, we correlated the methylation profile of each probe genome wide with BPT scores for DS participants only. A normal Q-Q plot of correlation coefficients revealed significant skewing from a normal distribution (Figure 3a). We used a Benjamini-Hochberg corrected cutoff p-value of 0.01 to determine significance, and a total of 79 probes met this criteria. To eliminate probes with very little difference between the DS participants or between DS and control participants, we filtered these for probes where the total range of methylation exceeded 10% within the DS participants and which passed a *t*-test for differences between DS and control at a p-value of 0.05, for a final total of 4 probes (Additional file 5: Table S5). Secondly, we performed the same correlation test on the 3300 DMPs from the previous section. With the same cutoffs, five probes were significantly correlated with BPT in DS participants, of which two had been identified in the first correlation analysis. Combining these two lists then results in seven probes that were both correlated with BPT in DS participants and significantly differently methylated between DS and control. Two of these were poorly correlated with BPT when all both DS and control samples were tested, leaving a total of five hits in four genes (Figure 3b-e, Additional file 5: Table S5). Two probes are found in a CpG island in the body of *TSC2*, which has been shown to be required for mTOR signaling in the brain, which has been associated with cognitive impairment [39,40]. The third is located 344 bp upstream of the TSS of *RND1*, a Rho GTPase on chromosome 12 that regulates axon extension in dendritic neurons [41,42]. The final two are located 1.1 kb upstream of the TSS of *KIAA1644*, an uncharacterized protein on chromosome 22, and one is 26 kb downstream of *BICC1*, involved in kidney development and located on chromosome 10. Since so few probes were found, functional enrichment analysis was not possible.

### Specific analysis of amyloid precursor protein (*APP*)

**Figure 4:**
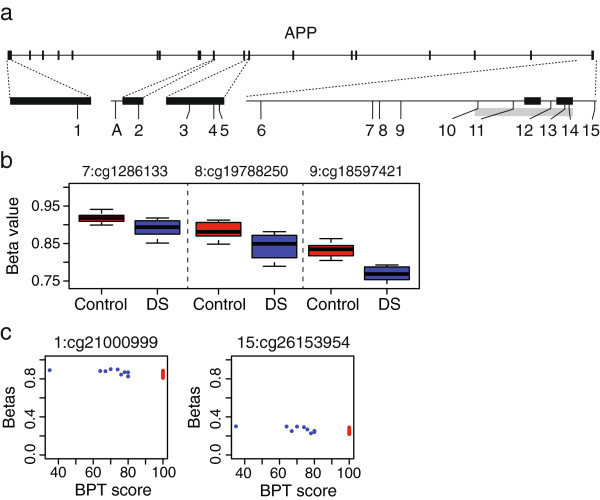
**Targeted analysis of *APP*. a)** Structure of the *APP* gene showing the locations of the 15 CpGs (numbers) and one CpH (letter A) analyzed in this study. Exons are shown as black boxes, intronic regions as lines. Grey box indicates position of the CpG island. **b)** Boxplots of three probes found to be significantly differently methylated between DS and control. CpG identification number as well as numbered position from A are indicated. **c)** Scatterplots of beta values (y-axis) versus Brief Praxis scores (X-axis) for the two probes which are significantly correlated with BPT. Blue points indicate DS participants and red points indicate controls.

No probes from *APP* were found to be significantly different between DS and control or correlated with BPT in the whole-genome analysis. To be sure that significant correlations were not merely being lost in the multiple testing correction, we performed a targeted analysis of the 15 CpGs and one non-CpG site from the array that localized to the *APP* gene (Figure 4a). We found that four CpGs were significantly differently methylated between DS and control (*t*-test p-value <0.05), one of which had less than 5% methylation in all samples, and the remaining three were all located in intron 1 (Figure 4b). This finding correlated with previous studies that showed hypomethylation of the promoter in DS patients [17]. We also found two CpGs correlated with BPT scores in DS participants only at a BH corrected p-value of 0.05, located on either end of the *APP* gene (Figure 4c). Both probes show higher methylation with higher BPT scores, which is consistent with the model that higher levels of *APP* are found in patients with AD, but the magnitude of difference between individuals is small and thus its biologically significance is unclear.

## Discussion and conclusions

Whole genome DNA methylation analysis of trisomies and cases of age-related cognitive impairment have revealed patterns of changes which appear to be associated with the etiology of each disease [18-20]. Here we add to the few studies examining whole-genome epigenetic perturbation in trisomies, and additionally show that this T21-related perturbation can coexist with an epigenetic signature associated with cognitive impairment. Comparing individuals with DS who show behavioural evidence of cognitive impairment to cognitively-normal age matched controls, we have found 3300 probes whose DNA methylation level differed by more than 10% between DS and control, and 5 probes which were correlated with Brief Praxis scores, a measure of cognitive impairment. Interestingly, however, two of the probes we found correlated with cognitive impairment in our DS participants were found in the *TSC2* gene, a component of the mTOR pathway that has been linked to Alzheimer’s Disease progression. Neither of these lists was enriched for sites on chromosome 21, and targeted analysis of the *APP* gene on chromosome 21 revealed weak if any evidence for epigenetic impacts on the gene hypothesized to cause the Alzheimer’s Disease-like phenotype often seen in people with DS. Particularly striking in this study was the fact that we found these signatures in buccal epithelial cells after correction for contaminating blood cells, highlighting both the pitfalls and potential rewards of using these easily-accessible tissues.

Comparing our results with previous studies examining DNA methylation in DS samples, we found an overlap in two of seven genes (*TSC2* and *DIO3*) that are differently methylated in fetal trisomy 21 skin and muscle, and five genes (*RYK*, *CASP10*, *MAP2K6*, *MSTR1*, and *RARA*) that are differentially methylated in trisomy 18 skin [19]. In another study using adult samples and parallel measurements of approximately 28,000 probes, 118 probes were found to be significantly different between the groups [18]. Interestingly, our data overlaps with 18 of the specific probes they found to be different in lymphocytes, and an additional 14 genes with different probes (Additional file 6: Table S6). For those probes which overlapped exactly, the direction of difference in DNA methylation between DS and control was the same in both studies [18]. A recent study used reduced representation bisulfite sequencing to examine DNA methylation in DS placenta. They found 629 sites in 597 unique genes that were significantly different between DS and control placenta. Of these, 93 genes were also found to differ between DS and control in our analysis, of which three *HOXA2*, *CPT1B*, and *GRM6* were found in all three studies (Additional file 7: Table S7) [18,20]. These sites must then become differently methylated in people with DS very early in development, since the placental tissue used in the latter study is of extraembryonic origin. It is reassuring that despite using different tissues and technologies, and considering that all three studies had relatively small cohorts, similar genes were found across studies. Also similarly to the two latter studies, we observed a bias towards hypermethylation in DS compared to control, with more than 66% of our significantly different probes being hypermethylated in DS [18,20].

Tissue composition differences are a bane of epigenetics studies, and thus it is important to continue to develop methods such as the one presented here to control for these differences. Signatures of different cell types, even within a given tissue, can easily mask true differences between groups [30,43], or, as we have shown, spuriously contribute to differences which may truly exist. Given that buccal swabs are a popular choice for population epigenetic studies because of their ease of collection and storage, it will be important for the research community to begin looking for and correcting these types of differences. A recent study examined buccal and blood whole-genome methylation, and determined that buccal cells cluster together with many other tissues, while blood methylation patterns are very distinct [44]. This makes correcting for potential blood contamination in buccal swabs especially important, since blood contamination would have a greater effect on buccal methylation profiles than other tissues. Our approach of using principal component analysis allowed us to robustly remove the variation caused by these tissue differences without removing entire probes or samples from the analysis. One potential problem is that we may be removing more variation than is required; if some true variation has the same projections as the tissue differences, we may be losing it as well. On the other hand, the current analysis gave very robust and unambiguous results after this correction, so any improperly lost variation would have had a minor contribution to the results.

The fact that an epigenetic signature that includes genes with functions related to the biology of DS is strong support for validity of our approach. The top four CpG clusters as mentioned in the Results section were related to diabetes, lipid metabolism, neural crest and craniofacial development, and connective tissue, all of which are connected to clinical features of DS. Our DAVID analysis further supported this, with enrichment clusters overlapping these functions as well as regulation of apoptosis and skeletal development. The massive enrichment for genes involving adhesion in our hit list for differentially methylated probes is interesting as, while a single CpG site in the Down Syndrome Cell Adhesion Molecule (*DSCAM*) gene is present on our hit list, there were 106 total adhesion-related genes in our DAVID hit list. *DSCAM* has been proposed to contribute to the congenital heart disease feature of DS, and it is possible that mis-regulation of a large number of adhesion-related genes through epigenetic modification may explain the increased risk in the DS population [45]. It is also notable that we found probes which differed between DS and control across all chromosomes. The lack of enrichment for changes in DNA methylation of probes on chromosome 21 is perhaps counterintuitive, despite the fact that it is supported by previous studies showing similar results [18,19]. It could be anticipated that having three copies of a chromosome would result in specific epigenetic modifications to attempt and control for dosage across the trisomy. Unfortunately the DS participants in our study were not karyotyped, so that we do not know the extent of their trisomy 21. Given the differences observed between DS and control, however, it is clear that the presence of at least a part of an extra chromosome 21 is capable of causing these genome-wide epigenetic changes.

Given that only five probes in four genes were found to be both correlated with BPT scores in DS participants and different between DS and controls, it is impossible to assess functional enrichment. Two of these four genes, however, have known functions in neural development or degeneration. *RND1* is a Rho GTPas that functions to promote dendritic cell growth and axon guidance, and loss of *RND1* expression suppresses axon formation in hippocampal neurons [41,42]. We found that methylation of *RND1* was positively correlated with BPT, meaning that lower levels of DNA methylation were associated with more severe cognitive impairment. If DNA methylation suppresses *RND1* expression, the opposite pattern might be expected in DS participants, but without knowing the mechanistic relationship between *RND1* expression and DNA methylation, it is difficult to interpret. The other gene, *TSC2*, had two CpGs in an island that overlaps with exons 27, 28, and 29 that showed decreasing DNA methylation with cognitive impairment. *TSC2* is involved in mTOR signaling, which regulates levels of tau in mouse neuronal models, resulting in neuropathological symptoms which are alleviated when mTOR signaling is reduced [39,40,46,47]. Levels of tuberin protein, the *TSC2* gene product, are decreased in brain samples of adult males with DS and AD-like symptoms, and a mouse knockdown of *Tsc2* showed an increase in tau-positive axon formation in hippocampal neurons [48,49]. In cancer cells, demethylation of the promoter of *TSC2* was shown to result in an increase in expression [50]. On the surface, this methylation data appears conflicting, however the island in which our two target CpGs are found is in the body of the gene, and island CpGs in gene bodies can be either positively or negatively correlated with gene expression [51]. We can therefore hypothesize that decreased methylation of *TSC2* in DS participants is associated with the decreased protein expression, which results in an increased probability of accumulation of tau through mTOR regulation, which predisposes these patients to AD-like disease. Thus *RND1* and especially *TSC2* are interesting targets for future studies of epigenetic alterations in cognitive impairment.

Cross-sectional studies such as these are important to discover associations between DNA methylation and cognitive impairment. Given our relatively small sample size, we were surprised to find such clear differences between our study groups, but in the future, it will be important to perform larger and more powerful longitudinal studies on patients with DS and AD to track the dynamics of DNA methylation changes during cognitive decline. Post-mortem brain tissue analysis would also shed further light on the relationship between epigenetics and cognitive decline in these patients. Together, these data will help illuminate whether DNA methylation is changed with cognition, whether altered DNA methylation is a predictive factor for cognitive decline, or whether the two processes occur simultaneously.

## Abbreviations

DS: Down syndome; AD: Alzheimer’s disease; GO: Gene ontology; BPT: Brief praxis test; PCA: Principal component analysis; DMP: Differentially methylated probe; DMR: Differentially methylated region; BH-DMP: Bump-hunter differentially methylated probe.

## Competing interests

The authors declare that they have no competing interests.

## Authors’ contributions

MJJ performed the data analysis and drafted the manuscript. PF performed the PCA and PC1 subtraction. KW collected the samples and performed the BPT assessment. SN, LMM, and JLH processed the samples, performed the arrays, and did preliminary data analysis. EE conceived the PCA analysis and assisted with statistical analysis. MC participated in the study design and edited the manuscript. NVB conceived the study and design, recruited the participants, and contributed to analysis and interpretation of the data. MSK conceived the study and design, and helped draft the manuscript. All authors read and approved the final manuscript.

## Pre-publication history

The pre-publication history for this paper can be accessed here:

http://www.biomedcentral.com/1755-8794/6/58/prepub

## Supplementary Material

Additional file 1: Table S1 Table of correlation coefficients and p-values for correlations of PCs after subtraction of PC1 and BPT scores or presence of DS. Click here for file

Additional file 2: Table S2 Table of CpG probes that are significantly differently methylated between DS and control participants. Click here for file

Additional file 3: Table S3 Table of CpG probes that are significantly differently methylated between DS and control participants and overlap with clusters found to be significantly differently methylated by Bump Hunter. Click here for file

Additional file 4: Table S4 DAVID results for probes that are significantly differently methylated between DS and control participants. Click here for file

Additional file 5: Table S5 Table of probes that are significantly correlated with BPT. Click here for file

Additional file 6: Table S6 Overlap of our DMPs with DMPs from the Kerkel study [18]. Click here for file

Additional file 7: Table S7 Overlap of our DMPs with DMPs from the Jin study [20]. Click here for file
